# Correlations between smartphone addiction and depressiveness, daytime sleepiness as well as per-ceived social support in adolescents

**DOI:** 10.1192/j.eurpsy.2023.826

**Published:** 2023-07-19

**Authors:** K. Rachubińska, A. M. Cybulska, D. Schneider-Matyka, M. Nowak, E. Grochans

**Affiliations:** Department of Nursing, Pomeranian Medical University in Szczecin, Szczecin, Poland

## Abstract

**Introduction:**

Background of behavioral addictions, which include smartphone addiction, is complicated and unclear. Belonging to an Internet community that shows many forms of virtual pathology and generates new, artificial trends also favors the development of mental implications which include lowered mental immunity, emotional lability, increased depressiveness, aggression, inadequate self-esteem, lowered self-value and personality disorders. Implications regarding the social sphere are above all related to the gradual alienation from the closest environment as a result of preoccupation with the subject of dependence. Those consequences include deterioration of social relations, loss of interpersonal skills and former interests, as well as the minimization of contacts with the closest ones which lead to the sense of social isolation and loneliness.

**Objectives:**

The aim of this study was to estimate the scale of mobile phone addiction among young adults as well as to establish whether the low level of perceived social support is related to the problematic smartphone use, and whether an addictive pattern of smartphone use is related to the prevalence of depressiveness and excessive daytime sleepiness.

**Methods:**

The study involved 567 young-adult respondents from West Pomeranian Voivodship in Poland. The study was carried out using the diagnostic poll method via questionnaire technique. Both the author’s own questionnaire and the following standardized research tools were used: the Mobile Phone Problem Use Scale for Adolescents (MPPUSA), the Beck Depression Inventory (BDI), the Epworth Sleepiness Scale (ESS), the Multidimensional Scale of Perceived Social Support (MSPSS).

**Results:**

Perceived social support was significantly lower in the group of respondents who problematically used their smartphones in comparison with the ones who used them in a correct way (p < 0.05). Severity of depressive symptoms and daytime sleepiness (p < 0.05) was significantly greater in respondents addicted to their mobiles in comparison with non-addicted ones.

**Conclusions:**

The scale of the mobile phone addiction phenomenon in respondents was low. Many respondents though expressed a subjective sense of being addicted to their smartphones in comparison with the obtained results. Problematic smartphone use concerns to the same degree members of both sexes, residents of villages and cities, as well as singles and ones in relationships (both formal and informal). The mobile phone addiction is associated with the risk of severe symptoms of depression and excessive daytime sleepiness. Pathologic pattern of smartphone use much more frequently concerned individuals who perceived their social support level as low.

**Disclosure of Interest:**

None Declared

